# Methylglyoxal, a Knot to Be Untied in Brain Glucose Hypometabolism

**DOI:** 10.3390/metabo15110690

**Published:** 2025-10-24

**Authors:** Vitor Gayger-Dias, Vanessa-Fernanda Da Silva, Thomas Michel Sobottka, Marina Concli Leite, Adriana Fernanda K. Vizuete, Carlos-Alberto Gonçalves

**Affiliations:** 1 Programa de Pós-Graduação em Bioquímica, Instituto de Ciências Básicas da Saúde, Universidade Federal do Rio Grande do Sul, Porto Alegre 90035-003, Brazil; vitor.dias@ufrgs.br (V.G.-D.); vanessa.fernanda@ufrgs.br (V.-F.D.S.); thomas.sobottka@ufrgs.br (T.M.S.); marina.leite@ufrgs.br (M.C.L.); adriana.vizuete@ufcspa.edu.br (A.F.K.V.); 2 Laboratório de Fisiologia Translacional, Programa de Pós-Graduação em Ciências da Saúde, Universidade Federal de Ciências da Saúde de Porto Alegre, Porto Alegre 90050-170, Brazil; 3 Programa de Pós-Graduação em Neurociências, Instituto de Ciências Básicas da Saúde, Universidade Federal do Rio Grande do Sul, Porto Alegre 90035-003, Brazil

**Keywords:** Alzheimer’s, astrocyte, diabetes mellitus, insulin resistance, methylglyoxal

## Abstract

**Background:** Advanced glycation end products (AGEs) and receptors for AGEs (RAGE) have been extensively implicated in metabolic and neurodegenerative disorders due to their capacity to alter protein structure and function through non-enzymatic glycation. More recently, methylglyoxal (MG), a highly reactive glycolytic byproduct, has gained attention as a critical mediator of AGE formation and an independent contributor to cellular distress, particularly in the context of diabetes mellitus and Alzheimer’s disease. **Objectives:** This review synthesizes evidence from experimental and clinical studies addressing MG generation and metabolism in brain tissue, emphasizing the glyoxalase system as the primary detoxification mechanism, the functional contribution of astrocytes, and the downstream consequences of MG accumulation. In addition, we examined the interplay between MG, RAGE signaling, unfolded protein response, and regulatory mechanisms involving the hexosamine biosynthesis pathway and O-GlcNAcylation of key proteins in glucose metabolism and insulin signaling. **Results and Conclusions:** Brain glucose hypometabolism is a consequence of insulin resistance and results in a metabolic rearrangement that expands the glycolytic pathway and generates more MG, which, in turn, can affect insulin signaling, further compromising the molecular basis of insulin resistance and creating a vicious cycle. Astrocytes are key cells in the generation and detoxification of MG in the brain, making them a therapeutic target.

## 1. Introduction

Methylglyoxal (MG) has gained prominence as a pivotal factor in the formation of advanced glycation end products (AGEs) and, consequently, the basis of ageing and prevalent neurodegenerative diseases, such as diabetic encephalopathy (DE) and Alzheimer’s disease (AD) [1,2]. We have highlighted the role of astrocytes in MG detoxification in vitro [3] and in vivo models of AD [4]. MG is increasingly being viewed as an undesirable side product of glucose metabolism.

Since MG is derived from glucose, exposure of cells to high levels of glucose commonly elevates MG levels, suggesting simply that this is due to high glucose and not the underlying insulin resistance. Herein, we review the insulin resistance as a mechanism behind MG elevation, as well as the MG detoxification pathway, its physiological and pathological effects, the pathophysiological conditions associated with its accumulation, and the prospects for its use as a marker in pathological conditions, always emphasizing the role of astrocytes in the generation and clearance of this compound.

## 2. Sources of Brain MG

**Figure 1:**
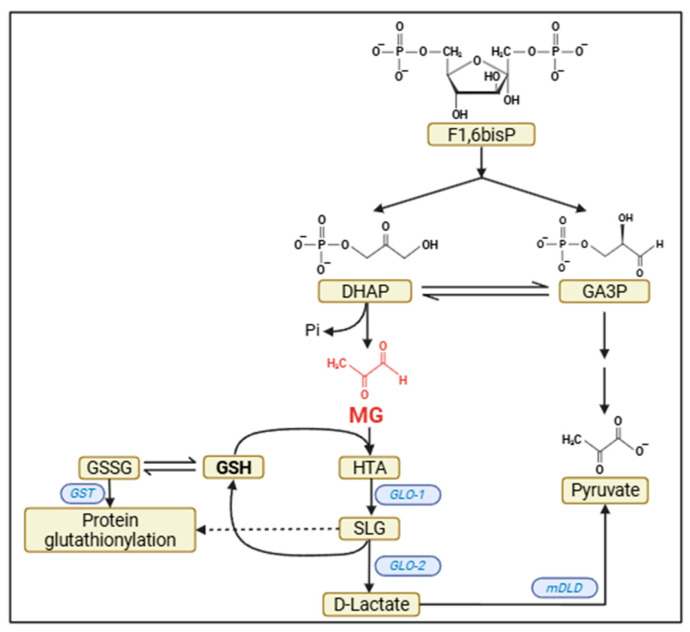
Origin and detoxification of MG. In the glycolytic pathway, fructose-1,6-bisphosphate (F1,6bisP) is cleaved to dihydroxyacetone phosphate (DHAP) and glyceraldehyde-3P (GA3P). Part of DHAP is spontaneously dephosphorylated and isomerized to methylglyoxal (MG). MG reacts with glutathione (GSH) to form hemithioacetal (HTA). HTA, in turn, in a reaction catalyzed by glyoxalase-1 (GLO-1), produces S-lactoyl-glutathione (SLG). SLG, in a reaction catalyzed by glyoxalase-2 (GLO-2), produces D-lactate and regenerates GSH. D-lactate is converted to pyruvate, delivering its electrons directly to the respiratory chain, in a reaction catalyzed by mitochondrial D-lactate dehydrogenase (mDLD). Note that oxidized glutathione (GSSG) can, spontaneously and through the action of GST (glutathione-S-transferase), glutathionylate -SH groups of proteins. SLG can also do this (as indicated by the dotted line), in a process catalyzed by GLO-2.

MG is 2-oxopropanal, a small cell-permeable compound that demonstrates high (glycating) activity with the amino groups of proteins and DNA [5]. This molecule is principally derived from the phosphotrioses of the glycolytic pathway (Figure 1); however, given that the ratio of the two phosphotrioses, dihydroxyacetone phosphate (DHAP)/glyceraldehyde 3-phosphate (GA3P) is 9/1, DHAP is the only metabolite frequently cited as a source of MG. The reaction is spontaneous and involves DHAP dephosphorylation and isomerization of glyceraldehyde to MG, but some bacteria possess an enzyme for this reaction, contradicting the idea that it is purely an accidental byproduct of metabolism. In addition to the phosphotrioses, there are two other minor sources of MG in mammals. The first is acetone, one of the ketone bodies, which can generate MG when oxidized by cytochrome P450 oxidase 2E1 (CYP2E1). This oxidase, which metabolizes ethanol to acetaldehyde, is therefore capable of generating MG under conditions of ketosis, such as fasting and diabetes mellitus (DM). The second source derives from threonine catabolism. Threonine is an essential amino acid that is metabolized to succinyl-CoA (mostly) or pyruvate. This second pathway can lead to the formation of aminoacetone, which, through the action of an amino oxidase, results in MG formation. It is estimated that physiologically, 0.1–0.4% of metabolized glucose results in MG [6]. However, less than 1% of the MG generated results in glycation, despite its high reactivity. More than 99% is detoxified by the glyoxalase system, which varies between tissues [2]. Thus, while we find approximately 4 μM of MG in the liver and muscle tissues, we find approximately 1 μM in brain tissue [7]. Levels of 10 μM are found in the extracellular medium.

Of note, the degradation of DHAP to MG is an uncatalyzed reaction, but that does not mean that it is constant. At least two metabolic variables affect this reaction: pH and the presence of reactive oxygen species (ROS). Since DHAP phosphorolysis begins with deprotonation (see [8] for a review), an alkaline pH would favor MG formation. Furthermore, if there is consumption of glutathione (GSH) (due to the presence of ROS) the GSH/GSSG ratio drops and this favors the glutathionylation of proteins, including GA3P dehydrogenase (GAPD), the enzyme in the reaction subsequent to the isomerization of phosphotrioses in the glycolytic pathway. Glutathionylation protects cysteine groups from ROS attack, but in the case of GAPD, there is also an inhibition of activity, retaining DHAP and favoring MG formation. Thus, under conditions of oxidative stress, there is greater MG formation. Commonly MG is seen as a glycotoxin, generated under conditions of oxidative stress; however, the concept of hormosis is worth mentioning, where a toxic substance can be protective at low concentrations (see [9] for a definition). Like ROS, physiological concentrations of MG stimulate the expression of metabolic pathway enzymes that protect cells from MG toxicity at higher concentrations, as will be discussed below.

## 3. Brain Detoxification

In the neuropil region, comparison of the cytosolic glycolytic pathways in neurons and astrocytes reveals stronger astroglial activity (see Barros et al., 2024 for a review) [10]. During neuronal activity, astrocytes provide lactate to sustain this activity. This more vigorous glycolytic activity results in greater MG production, which also explains the greater MG detoxification activity in astrocytes, primarily through the glyoxalase system [11].

The system is composed of two enzymes, glyoxalase 1 and 2 (GLO 1 and 2, respectively) (Figure 1). Although the enzymes are ubiquitous, studies in primary cortical cultures indicate that GLO-1 is almost 10 times more active in astrocytes than in neurons, while GLO-2 is twice as active [12]. In the first, uncatalyzed reaction, MG conjugates with GSH, forming hemithioacetal, which is then converted into S-lactoylglutathione by the catalytic action of GLO-1. This compound is subsequently cleaved by the action of GLO-2 to D-lactate, regenerating GSH. The reaction catalyzed by GLO-1 is the rate-limiting step in the detoxification process. Considering the greater activity of the glyoxalase system in astrocytes, as well as their ability to generate and recycle GSH, the importance of these cells in MG clearance in brain disorders is unquestionable [2,11]. It is worth noting that GLO-2, from S-lactoylglutathione, can catalyze the protein glutathionylation previously described [13].

The D-lactate generated can be transported to mitochondria through a D-lactate/H^+^ symport and a malate antiport. The incoming D-lactate is oxidized to pyruvate by D-lactate dehydrogenase, a mitochondrial flavoenzyme. Thus, the electrons from this reaction go directly to the respiratory chain. The outgoing malate also generates pyruvate, through the action of the malic enzyme, and the electrons generate NADPH, which can potentially be used in the antioxidant response [13].

## 4. Extracellular MG Signaling via RAGE

The discovery of AGEs and signaling via the receptor for AGEs (RAGE) has expanded our understanding of many chronic diseases, particularly DM [14] and neurodegenerative diseases such as Alzheimer’s dementia [15]. Despite the denomination of RAGE, it is not a specific immunoglobulin receptor for AGEs, and also binds, at different sites, lipopolysaccharide (LPS) from Gram-negative bacteria, S100 calcium-binding protein B (S100B), and amyloid beta peptide from neurons [16,17]. This receptor, when activated, mobilizes pathways such as nuclear factor kappa B (NFkB) and mitogen-activated protein kinase (MAPK), which are sensitive to oxidants, and NADPH oxidase (NOX), which generate superoxide and intercommunicate, triggering an inflammatory response. If this inflammatory response occurs chronically, due to a high production of AGEs and/or lack of their clearance, cell death processes are induced [4,18,19].

Evidence increasingly indicates that MG is the main generator of AGEs due to its permanent endogenous production, its permeability and its high reactivity with amine groups in proteins and DNA [15]. Thus, the extracellular effects of MG, primarily mediated by RAGE, have been widely investigated both in vivo and in vitro, either directly using MG exposure or indirectly through exposure to high glucose levels. Notably, MG has stronger glycating activity than glucose; however, while glucose is present in the extracellular medium at mM concentrations, MG is found only at μM concentrations. This indicates that the reactivity of MG (preferentially for arginine) is thousands of times greater than that of glucose, which preferentially targets lysine [20].

Several studies, in vitro and in vivo, have characterized the neuroinflammatory changes that are induced by increased extracellular MG [21,22] as well as changes in neurotransmission [23], blood–brain barrier permeability [20,24], generation of amyloid beta peptides [25] and beta amyloidogenesis [26], which may underlie behavioral changes and cognitive impairments [27,28]. However, it is important to note that the effects of MG may also be RAGE-independent [29,30] and that GABA_A_ receptors have more recently been shown to be direct targets of modulation by MG [31,32].

## 5. Direct Intracellular Consequences of Increased MG in the UPR

In addition to the formation of extracellular AGEs, intracellular MG directly affects protein machinery, including the machinery involved in protein homeostasis, causing and/or aggravating the unfolded protein response (UPR) (see [33,34] for reviews on UPR in the CNS). Cellular protein homeostasis depends greatly on the endoplasmic reticulum (ER), which involves, in addition to protein synthesis, protein folding (mediated by chaperones), co-translational modifications and even degradation in cases of misfolding [35]. This operational flow is modulated by various nuclear and cytosolic factors, through proteins in the membrane ER proteins called sensors. These sensors trigger the UPR, which is essentially an adaptive response; however, if prolonged, it can lead to cell death. Furthermore, persistent UPR appears to be the basis of neurodegenerative diseases.

Three classes of sensor proteins have been characterized: PERK (PKR-like ER kinase), IRE1α (inositol-requiring transmembrane kinase/endoribonuclease 1α), and ATF6 (activating transcription factor 6). MG, which escapes detoxification mechanisms, reacts predominantly with arginine (forming hydroimidazolones), but can react to a lesser extent with lysine. This glycation alters the charge and hydrophobicity of the protein chain, potentially leading to misfolding [34]. Protein misfolding in the ER triggers the UPR, which ultimately results in reduced protein synthesis, but also in the clearance of misfolded proteins and the increased expression of “restorative proteins”, such as chaperone proteins or proteins protective against oxidative stress, reticular calcium leak, or glucose starvation.

Studies on the direct effects of MG on human lens epithelial cells have characterized the activation of UPR by oxidative stress and increased reticular calcium release. With time, these effects cause a reduction in sensor proteins, providing insights into cataract formation in diabetic patients [36]. A more recent study with human aortic endothelial cells reinforces the activation of the UPR by MG, as well as the key role of GLO-1 in cellular defense [37].

GLO-1 expression is regulated by nuclear factor erythroid 2-related factor 2 (Nrf2) and NFkB. Under basal conditions, Nrf2 is ubiquitinated and undergoes proteasomal degradation dependent on KEAP1, a ROS-sensitive protein. Under oxidative stress, Nrf2 is released from KEAP1 and migrates to the nucleus, increasing the expression of antioxidant proteins such as glutamate–cysteine ligase (GCL), the rate-limiting enzyme for GSH synthesis. Interestingly, MG also acts as a crosslinker, forming mercaptomethylimidazole between thiol (cysteine) and amine (arginine) groups (MICA) [20]. When KEAP1 has MG-induced MICA bridges, it also releases Nrf2, increasing the expression of antioxidant enzymes [38]. It is not yet known which other UPR proteins may undergo MICA-induced modifications by MG.

In addition to KEAP1 regulation, Nrf2 is modulated by phosphorylation (see [39] for a review). For example, phosphorylation by PKC at Ser^40^ or by AMPK at Ser^550^ increases Nrf2 stability and activity, respectively. In contrast, phosphorylation by glycogen synthase kinase 3β (GSK-3β) at Ser^335^ facilitates its ubiquitination and degradation. PERK also positively modulates Nfr2 expression in the UPR. However, under conditions of prolonged UPR stimulation, calpain 1/2 could lead to the cleavage of p35, a positive modulator of cyclin-dependent kinase 5 (CDK5), which also positively modulates Nrf2. In other words, calpain would lead to a reduction in Nrf2 activity and stability. This mechanism involving calpain activation, which has been hypothesized to occur particularly in astrocytes, would explain the reduction in Nrf2 under conditions of chronic UPR activation, as found in neurodegenerative diseases [40,41].

## 6. But What Is the Knot in the MG Metabolism?

Cells exposed to high glucose levels, whose glucose entry is independent of insulin, are commonly considered permeable to glucose and, therefore, generate MG through the glycolytic pathway. However, in DM and AD, there is no glucose hypermetabolism with uncontrolled glucose entry; instead, glucose hypometabolism occurs due to insulin resistance, as shown in vivo by reduced glucose transport (and, indirectly, reduced metabolization) [42]. As such, elevated MG is now viewed both as a consequence and also a cause of insulin resistance.

But how can we explain the increase in MG in glucose hypometabolism? To understand the increase in MG, we need to examine other pathways of glucose metabolism, particularly in astrocytes (see [43] for a review). Glucose is not only an immediate fuel for brain tissue, but it is also storable (as glycogen) and serves as a substrate for neurotransmitter synthesis (glutamate, GABA), NADPH for GSH recycling and lipid synthesis, and as a carbon source for membrane lipids (cholesterol and phospholipids). We need to understand that these pathways are reduced due to insulin resistance, and that this redistribution favors the glycolytic/lactogenic pathway, increasing MG formation.

Note that astrocytes exhibit broad morphological and functional heterogeneity, which is even greater than that of neurons [44]. Furthermore, it is important to remember that the various studies show snapshots of the reality of AD and DE, whose manifestations are a continuum, reflecting temporal (i.e., a given parameter may be elevated at one time and reduced at another) and regional (i.e., a given marker may be elevated in one brain region and reduced in another) metabolic heterogeneity.

**Figure 2:**
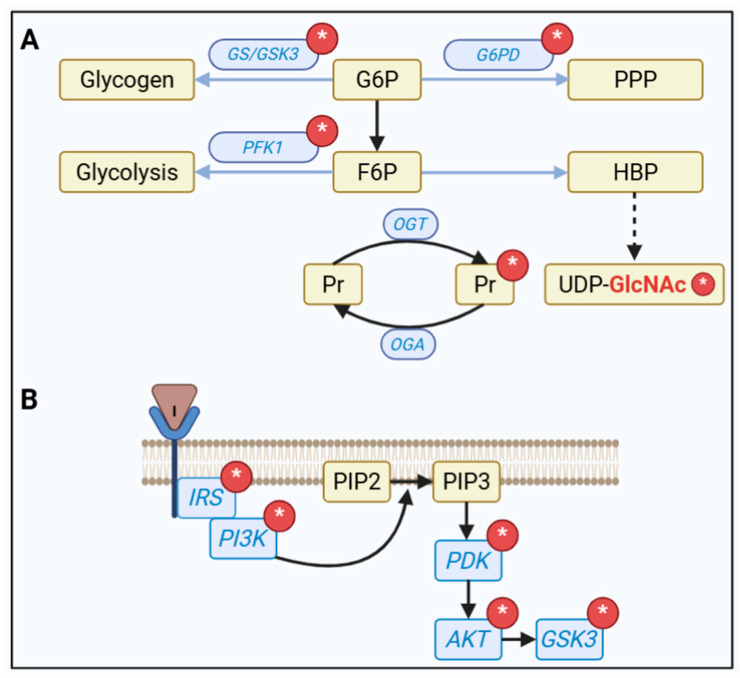
Regulation of glucose metabolism pathways and the insulin pathway by NAGylation. (**A**) Glucose-6P (G6P) fates: glycogen synthesis and pentose phosphate pathway (PPP); Fructose-6P (F6P) fates: glycolysis to pyruvate/lactate and hexosamine biosynthesis pathway (HBP), where UDP-GlcNAc is synthesized. The cycle between the N-acetylglucosamine transferase (OGT) enzyme that adds NAG (represented by an asterisk in a red circle) and the O-GlcNAcase (OGA) enzyme that removes NAG from proteins is shown below. Key regulatory enzymes in these pathways regulated by NAGylation are indicated: glycogen synthase (GS), glycogen synthase kinase 3 (GSK3), glucose -6-phospate dehydrogenase (G6PD), and phosphofructokinase 1 (PFK1). (**B**) Key proteins of the insulin signaling pathway that are regulated by NAGylation (represented by an asterisk in a red circle): insulin receptor substrate (IRS), phosphatidyl inositol 3 kinase (PI3K) and PIP3 dependent kinase (PDK).

At the beginning of both AD and/or ED, oxidative distress may demand a greater flow of the pentose phosphate pathway (PPP) to produce and recycle GSH (see Figure 2). However, in more advanced stages, this system may be compromised, as indicated by reduced activity of glucose-6-phosphate dehydrogenase (G6PD) [45]. On the other hand, glycogen synthesis may be reduced from the onset of AD, possibly due to an alteration in the AKT/GSK-3 communication of insulin signaling, and this per se contributes to cognitive alterations, with impairment of learning and memory [46].

In addition to the glycolytic pathway and the two routes mentioned above, the hexosamine biosynthesis pathway (HBP), although a quantitatively small destination, has a regulatory effect on the other pathways. The glucose pathway that produces UDP-GlcNAc, used for post-translational modification of O-GlcNAcylation (or simply NAGylation) on Ser or Thr residues [43], is also reduced in DM and AD, possibly reflecting the lower availability of glucose [47]. However, we know relatively little about the diverse significance of specific protein modifications by NAGylation and the crosstalk with phosphorylation, particularly in brain tissue. This is an important aspect for consideration, especially given our observation, in a model of dementia with streptozotocin, that the reduction in NAGylation apparently precedes insulin resistance, as indicated by the phosphorylation of IRS-1 at Ser^309^ [48]. Indeed, several enzymes in the insulin signaling pathway and key enzymes for glucose metabolization pathways, such as glycogen synthase, glucose-6P dehydrogenase and phosphofrutokinase-1, are NAGylated (see Figure 2).

As a consequence, changes in intracellular glucose flux result in increased glycolytic activity with increased lactate and MG production. Accordingly, AD patients demonstrate increased lactate in their cerebrospinal fluid, as well as a reduction in pyruvate levels [49]. Notably, pyruvate is also used to produce cholesterol for exportation to neurons. Although we know that increased neuronal cholesterol worsens disease progression, it seems logical that this is due to the neuron’s inability to perform clearance (via cytochrome P450 46A1), and not to the increase in astroglial cholesterol synthesis [50]. In summary, the greater glycolytic flux (in this hypometabolic condition) may also result in greater MG production, as confirmed by the increased activity of the glyoxalase system [51].

## 7. Pharmacological and Nonpharmacological Strategies for Controlling MG

Given the central role of the central glyoxalase system in MG detoxification, with GLO-1 as the rate-limiting step, pharmacological neuroprotective strategies focus on GLO-1 activators (by increasing the activity and/or expression of the enzyme) and scavengers that complement the antioxidant activity of the system. Drugs such as metformin [52,53,54], aminoguanidine [55,56,57], simvastatin [58], and glucagon-like peptide-1 agonists [59,60,61] have been identified as GLO-1 activators. Scavengers include resveratrol [62,63,64], sulforaphane [65,66], curcumin [67], glycine [68,69], pyridoxamine [70] and flavonoids such as hesperetin [71,72] and quercetin [73,74]. It is worth noting that most studies with these compounds did not directly evaluate the expression, content, or activity of GLO-1 in neural cells. However, quercetin positively modulated GLO-1 in both cerebellar neuronal culture [73] and in the cerebral cortex of STZ-induced diabetic rats [74].

Nonpharmacological approaches, such as physical exercise and diet, modulate energy metabolism and antioxidant defense. Physical exercise has been identified as a modulator of Nrf2, increasing GSH and GLO-1 levels and contributing to MG detoxification [75]. A study with mice subjected to treadmill exercise observed a direct increase in the GLO-1 and GLO-2 enzymes in the cerebral cortex [76] and GLO-1 in the hippocampus [77]. In humans, after running exercise, a decrease in MG levels and an increase in D-lactate levels in the blood were observed [78]. With regard to diet, caloric restriction and ketogenic diets are also reported to increase the expression of Nrf2 [79,80].

## 8. Final Comments and Perspectives

There is no doubt that insulin resistance, as observed in metabolic syndrome, accompanies and/or underlies DE and AD [81]. Note that insulin resistance is also observed in major depressive disorder [82], another condition considered to be a risk factor for AD.

Glucose hypometabolism is a consequence of insulin resistance and results in a metabolic rearrangement that expands the glycolytic pathway and generates more MG. MG, in turn, can affect insulin signaling, further compromising the molecular basis of insulin resistance and creating a vicious cycle. Astrocytes are key cells in the generation and detoxification of MG in the brain, making them a therapeutic target.

Although several biomarkers have been proposed for monitoring the course of DM, fasting plasma glucose and glycated hemoglobin (HbA1c) are still the biomarkers of choice for DM. However, considering the limitations of using HbA1c in cases of low Hb (anemia) or accelerated red blood cell turnover (due to chronic kidney disease), MG, due to its close relationship with insulin resistance, must be considered as a potential diagnostic and prognostic marker, not limited to DM [83]. However, methodological approaches and measurement variability are still important difficulties [84].

MG, due to its high capacity to generate AGEs (sometimes referred to as gerontoxins) [85] and the activation of the UPR, is a key element in the basis of neurodegenerative diseases and aging. Taken together with the data available on its production and detoxification, we propose that while under physiological conditions, MG may stimulate antioxidant defenses via Nrf2, chronic exposure may lead to continuous activation of the NFkB pathway, both intracellularly via the UPR, and extracellularly via RAGE.

Finally, more recently, the direct effects of MG on GABA receptors [86] and the dopamine pathway [27] have been proposed and investigated, which could justify the alterations observed in neuropsychiatric disorders, and may be independent of a degenerative basis.

## Data Availability

The raw data supporting the conclusions of this article will be made available by the authors on request.

## Abbreviations

The following abbreviations are used in this manuscript:

AD	Alzheimer’s disease
AGEs	advanced glycation end products
ATF6	activating transcription factor 6
CDK5	cyclin-dependent kinase 5
CYP2E1	cytochrome P450 oxidase 2E1
DE	diabetic encephalopathy
DHAP	dihydroxyacetone phosphate
DM	diabetes mellitus
G6PD	glucose-6-phosphate dehydrogenase
GA3P	glyceraldehyde 3-phosphate
GAPD	glyceraldehyde 3-phosphate dehydrogenase
GCL	glutamate-cysteine ligase
GLO1	glyoxalase 1
GLO2	glyoxalase 2
GSH	glutathione
GSK-3β	glycogen synthase kinase 3β
HBP	hexosamine biosynthesis pathway
IRE1α	inositol-requiring transmembrane kinase/endoribonuclease 1α
LPS	lipopolysaccharide
MAPK	mitogen-activated protein kinase
MG	methyliglyoxal
NFκB	nuclear factor kappa B
NOX	NADPH oxidase
Nrf2	nuclear factor erythroid 2-related factor 2
PERK	PKR-like ER kinase
PPP	pentose phosphate pathway
RAGE	advanced glycation end products receptor
ROS	reactive oxygen species
S100B	S100 calcium binding protein B
UPR	unfolded protein response
